# Three-Dimensional Architecture of Placental Villous Branching in the Normal Human Placenta: A Scoping Review

**DOI:** 10.7759/cureus.107004

**Published:** 2026-04-14

**Authors:** Natalia Sinou, Amir Shihada, Alexandros Samolis, Nikoleta Sinou, Theodore Troupis, Dimitrios Filippou

**Affiliations:** 1 Department of Anatomy, National and Kapodistrian University of Athens School of Medicine, Athens, GRC

**Keywords:** 3d anatomy, branching, human, normal, placenta, placental, villous

## Abstract

The human placenta is a meticulously structured organ, whose functional effectiveness is contingent on the spatial and microanatomical arrangement of the chorionic villi's tree-like network. Recent advancements in three-dimensional (3D) imaging techniques have enabled in-depth examination of the hierarchical structure, vascularization, and branching patterns of these villi. This progression has facilitated the correlation of structural characteristics with the functionality of utero-fetal exchange.

The purpose of this scoping review is to systematically map and synthesize the existing literature on the 3D architecture and branching patterns of chorionic villi in the normal human placenta.

A comprehensive investigation was carried out using PubMed and Scopus databases, yielding 11 and 295 articles, respectively, from which 40 were deemed pertinent for this study.

The available evidence indicate that the villous tree in a normal human placenta presents a highly structured hierarchy, established early in gestation. The terminal villi possess intricate capillary networks characterized by loops, anastomoses, and fixed angles of branching, all of which minimize diffusion distances and enhance blood flow. The close spatial proximity of vessels, stroma, and syncytiotrophoblast not only provides mechanical stability but also facilitates effective gas and nutrient exchange.

The 3D configuration of placental villi forms a functionally optimized, hierarchical system that meets the hemodynamic and metabolic requirements of pregnancy. The deployment of advanced imaging and morphometric techniques has transitioned placental research from solely anatomical description to more mechanistic and predictive frames.

## Introduction and background

The placenta's significance is mainly determined by the exact spatial arrangement and microanatomical structure of the villous architecture, which is essential for accelerating the exchange of nutrients, gases, and metabolites between the mother and the fetus [1]. The villous tree is structured in a hierarchical manner into stem villi, intermediate villi, and terminal villi, with each category showcasing distinct morphometric, vascular, and functional features. Stem villi provide the main structural support and contain larger vessels, intermediate villi represent transitional branching forms, and terminal villi are the primary sites of maternal-fetal exchange, characterized by thin trophoblastic layers and dense capillary networks. These components work together to create the fundamental framework for interactions at the feto-maternal interface [2]. The complex branching patterns, microvascular topology, and three-dimensional (3D) structure of the placental villous networks are difficult for conventional histopathological techniques like two-dimensional (2D) light microscopy and standard hematoxylin-eosin staining to accurately depict. This abstracts thorough analyses of structural-functional correlations [3].

The precise evaluation of villous structures, capillary density, and branching patterns has been greatly facilitated by advances in high-resolution imaging techniques, including confocal laser scanning microscopy, multiphoton microscopy, 3D stereology, and volumetric light sheet imaging [4]. By enabling spatially specified reconstructions of placental microanatomy, these methods show the villous trees as hierarchical, intricately structured, and functionally optimal systems [5]. Quantitative morphometric analysis has converted contemporary understanding of placental deconstruction, establishing a connection between structural association and physiological effectiveness, along with pathophysiological variations in maternal-fetal exchange mechanisms [6]. Furthermore, the applicability and resolution of 3D imaging techniques may vary depending on the gestational age at which they are performed. Early gestation studies (first trimester) primarily capture the initial formation and branching of the villous tree, whereas second and third trimester analyses provide more detailed insights into terminal villi differentiation, capillary network maturation, and functional specialization.

Predictive assessments of placental function can now be accomplished by combining computational modeling with 3D morphometric and vascular mapping, associating changes in villous branching, intervillous perfusion, and capillary arranging with clinical issues such as intrauterine growth restriction, preeclampsia, and various placental insufficiency syndromes [7,8]. As a result, these developments not only improve anatomical standards but also provide a strong basis for translational research aimed at comprehending placental pathophysiology and developing therapeutic intervention approaches [9].

## Review

Materials and methods

**Figure 1:**
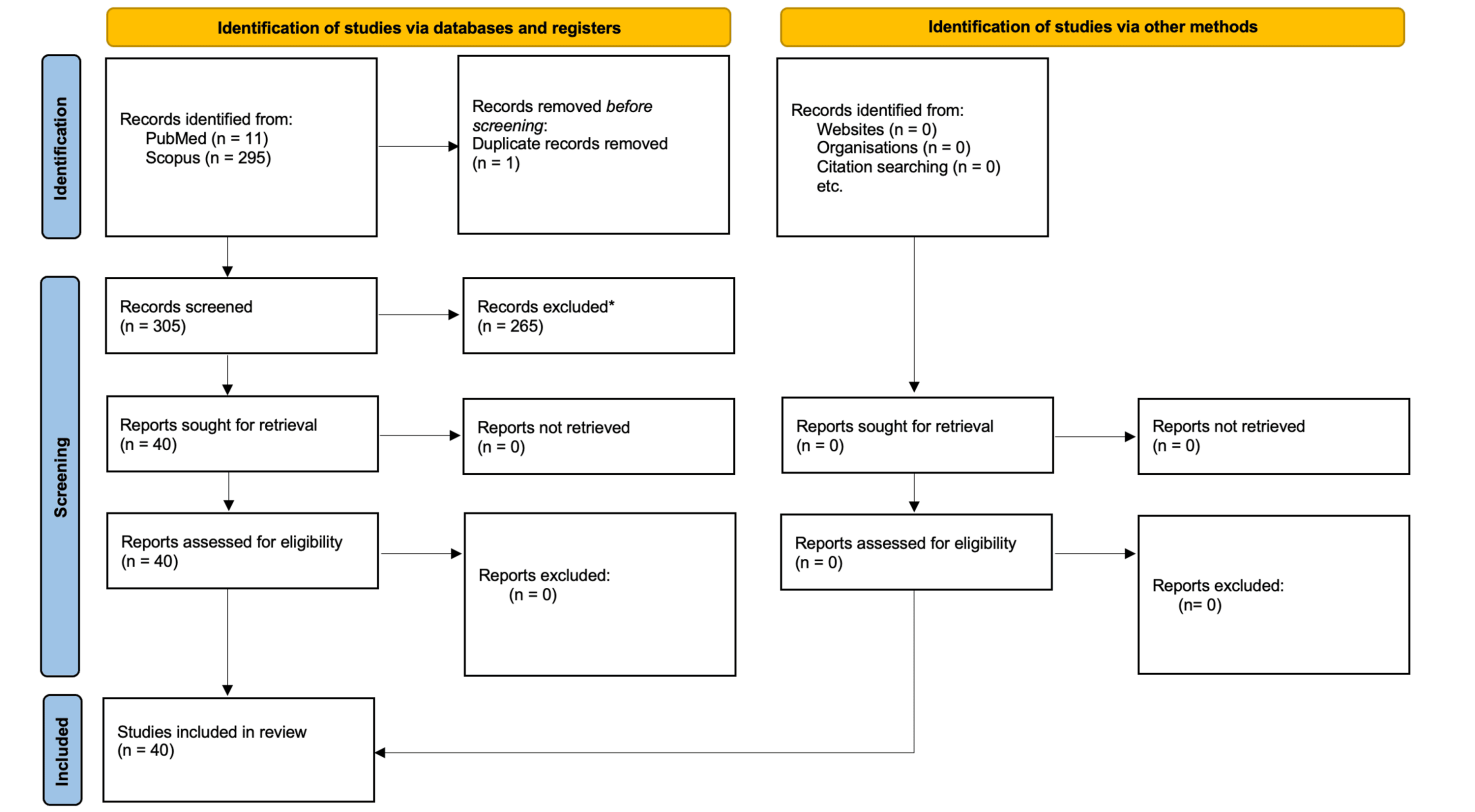
PRISMA flow diagram *title, abstract, or full text non-relevant PRISMA: Preferred Reporting Items for Systematic Reviews and Meta-Analyses

This study was conducted as a scoping review in accordance with the Preferred Reporting Items for Systematic Reviews and Meta-Analyses extension for Scoping Reviews (PRISMA-ScR) guidelines. A comprehensive review of the existing bibliography was performed utilizing the PubMed and Scopus databases. The search employed keywords including "three dimensional", "anatomy", "placental villous branching", and "normal human placenta". The search was conducted up to February 2026. Data were gathered using a standard data extraction form in line with these keywords. Studies were included if they (1) investigated the 3D structure of placental villi, (2) focused on normal human placentas, and (3) used imaging, morphometric, or computational analysis methods. On the other hand, studies were excluded if they (1) focused exclusively on pathological placentas without reference to normal structure, (2) were non-English, and (3) were reviews, editorials, or case reports without primary structural data. Initially, the PubMed search yielded 11 records, while Scopus provided 295. One duplicate was identified and removed. A total of 265 articles were excluded due to irrelevant titles, abstracts, or full texts. Ultimately, 40 references met the specified criteria and contributed to this review (Figure 1). The extracted data were analyzed qualitatively and synthesized descriptively, focusing on the hierarchical organization, 3D structure, and functional implications of placental villous branching. All screening and data extraction steps were performed by all authors.

Results

Hierarchical Structure of the Villous Tree

High-resolution confocal laser scanning microscopy and stereological analyses have repeatedly depicted that the placental villous tree presents a meticulously organized hierarchical architecture, which is established early in pregnancy [10]. The vascular development of chorionic villi produces a systematic, increasingly branching network instead of a chaotic capillary mesh, indicating precise morphogenetic control, according to studies conducted on placentas during the first trimester [4]. The primary structural support is provided by stem villi, which additionally serve as the spatial template from which intermediate and terminal villi develop and differentiate [11]. By establishing a multilayer terminal circulatory that supports both large-scale and small-scale perfusion dynamics, this hierarchical architecture improves maternal-fetal exchanges [12].

3D Configuration of Terminal Villi

The extensive intravillous capillary networks of terminal villi have been visible by sophisticated 3D reconstruction techniques revealing complicated anastomotic routes, elongated capillary loops, and sinusoidal dilation. Jirkovská et al. [1] indicated that these capillary loops frequently travel in eccentric pathways across the villous stroma. In addition, several studies have reported quantitative associations between villous branching configuration and capillarization indices. Increased capillary density, capillary volume, and exchange surface area have been observed in more extensively branched terminal villi. Stereological analyses further indicate that these parameters are closely related to reduced diffusion distances and enhanced efficiency of maternal-fetal exchange, highlighting the functional relevance of the observed 3D architecture. Moreover, several studies have reported details on the angles of branching and the overall geometric arrangement of terminal villi, highlighting a systematic, non-random organization that likely optimizes perfusion and exchange efficiency. The efficacy of transplacental exchanges is ameliorated by this design, which significantly decreases the diffusion distance between the fetal capillaries and the surrounding syncytiotrophoblast layer [13]. The angles of villous branching are continuously maintained, according to additional quantitative 3D microscopic morphometry, suggesting a systematic geometric layout as opposed to a random arrangement [14]. Such preserved topology indicates the branching structure's biomechanical and hemodynamic optimization, which may have an impact on capillary shear stress, intervillous flow, and the kinetics of nutrient transport [5]. The delicate relationship between the villi's morphology, the stroma's composition, and their functional efficiency at the maternal-fetal interface is highlighted by the terminal villi's spatial consistency [15,16].

Capillary Configuration and Spatial Arrangement

Topological analyses of villous capillaries have demonstrated a highly linked network with multiple bifurcations and sporadic anastomotic connections, resulting in a microvascular design that ensures equal perfusion across terminal villi [6]. This capillary structure minimizes hypoperfusion areas and promotes uniform gas and nutrient apportionment within the villous parenchyma, as indicated by stereological studies [17,18]. Advanced ultrastructural 3D analyses have shed light on the spatial relationships between villous capillaries and stromal elements [16]. The vascular tree's axis determines how collagen fibers are aligned, providing it with mechanical integrity and adaptability to fluctuating hemodynamic pressures [19]. Specifically, both capillary segments and stromal components have been reported to exhibit a preferential alignment parallel to the longitudinal axis of the vascular tree. This organized orientation supports efficient blood flow distribution and contributes to the mechanical stability of the villous structure under varying hemodynamic conditions. Some studies have further proposed underlying mechanisms for this organized alignment, suggesting that hemodynamic forces such as shear stress and blood flow patterns, as well as biomechanical interactions within the villous stroma, play a key role in shaping vascular orientation and branching behavior. These mechanisms are thought to contribute to the optimization of both structural stability and functional efficiency of the placental microcirculation. This orientation appears to be essential for preserving structural integrity while enabling the intervillous area to adjust nimbly to alterations in the maternal blood flow [7,20].

Ultrastructural Characteristics of Villous Branching

Significant insights into the microanatomical foundation of branching and the ultrastructural characteristics of villous architecture have been procured by both transmission and scanning electron microscopy [21]. Localized syncytiotrophoblast layer thinning and a decline in stromal density are typical characteristics of villous bifurcation sites; these alterations reduce diffusion barriers and improve exchange efficiency [8,21]. The nuclei of syncytiotrophoblasts are distributed in a non-random manner, creating spatially coherent patterns that correspond with branching structure and villous geometry, according to recent cytological imaging analyses. This nuclear organization presumably reflects mechanical transudative signaling pathways that control vascular remodeling, villous development, and differentiation, directly relating cellular architecture to transplacental transport functional effects [9].

Methodological Insights From Comparative 3D Studies

Although pathologically altered placentas constituted the focus of multiple initial 3D analyses, these studies have yielded useful analytical frameworks applicable to normal villous anatomy [22,23]. Indirectly highlighting the highly regulated structural arrangement of typical villi, 3D reconstructions of placentas damaged by maternal diabetes, for instance, revealed abnormalities in capillary branching, lumen size, and vascular connections [10]. These abnormalities were characterized by reduced branching complexity, irregular capillary looping, altered vessel diameters, and disrupted vascular connectivity. In addition, some studies reported decreased capillary density and uneven spatial distribution of vessels, which may affect perfusion efficiency. Similarly, assessments of growth-restricted placentas using volumetric stereology and computational modeling also established reliable benchmarks for comparative morphometric evaluations by validating quantitative techniques to examine villous branching architecture, capillary density, and perfusion topology [11,24-26].

In sum, these advanced imaging and analytical approaches have significantly amended the understanding of the human placental villous tree as a hierarchically structured, 3D optimized system [27]. They forge an essential link between microstructural arrangement, functional performance, and pathological alterations, laying a solid root for translational research in placental growth, maternal-fetal exchange physiology, and the pathophysiology of gestational conditions [28,29]. 

Discussion

This discussion synthesizes the current evidence on the 3D architecture of placental villous branching, highlighting structural patterns, functional implications, and areas where further research is needed.

The reviewed evidence indicates that human placental villi follow consistent 3D anatomical and morphometric principles. The hierarchical organization of the villous tree appears finely tuned to optimize exchange surface area and ensure effective perfusion throughout the terminal villi. Quantitative analyses of branching geometry, capillary looping patterns, and spatial arrangement of stromal and vascular components support this view [3,30,31]. These structural patterns facilitate the balanced distribution of nutrients and gases to the fetus while providing the mechanical robustness needed to withstand maternal blood flow fluctuations.

The stem villi provide the primary structural framework and serve as the template for branching; the intermediate villi act as conduits for maternal-fetal exchange while supporting vascular development; and the terminal villi, with their dense capillary networks, maximize the surface area for nutrient and gas exchange [1-5].

A tightly regulated developmental program is reflected in consistent branching angles, capillary trajectories, and topological configurations preserved across gestational stages [32]. These enduring architectural features suggest that the villous tree can dynamically adapt to the rheological constraints of intervillous blood flow and biomechanical stresses, such as shear stress and intravillous pressure gradients [6]. In this context, the villous architecture functions as an integrated structural and functional network, responding actively to hemodynamic, physiological, and mechanical demands [33].

Advances in modern 3D imaging techniques, including confocal laser scanning microscopy, multiphoton imaging, volumetric light sheet microscopy, and stereological reconstruction, combined with quantitative morphometric and topological analyses, have been key in revealing these insights [7,34,35]. This methodological progression marks a shift from descriptive histology toward a mechanistic understanding of placental microarchitecture [36]. Consequently, current studies establish direct links between villous microstructure and functional efficiency, providing a framework to explore pathological conditions such as intrauterine growth restriction, preeclampsia, and other forms of placental insufficiency, alongside normal maternal-fetal exchange [7,37].

Overall, 3D analyses demonstrate that the human placenta is hierarchically organized, biomechanically sophisticated, and functionally efficient, with villous branching patterns that are both structurally conserved and adaptively responsive to intrauterine conditions [38]. These findings underscore the importance of integrating spatial microanatomy, vascular topology, and stromal organization to understand placental function and the mechanisms underlying disease-related structural alterations [8].

A summary of the main characteristics and findings of the included studies is presented in Table 1.

**Table 1:** Summary of selected studies on 3D placental villous architecture IUGR: intrauterine growth restriction; 3D: three-dimensional

Author (year)	Sample type	Gestational stage	Imaging technique	Key findings	Relevance to normal placenta
Jirkovská et al. (2012) [1]	Human placenta	Term	Confocal microscopy	Capillary looping and spatial arrangement	Describes normal terminal villi microvasculature
Haeussner et al. (2014) [4]	Human placenta	Variable	3D light microscopy	Consistent branching angles and geometry	Supports structured villous hierarchy
Lisman et al. (2007) [5]	Human placenta	First trimester	Confocal microscopy	Early vascular branching patterns	Demonstrates early villous development
Vizza et al. (2005) [6]	Human placenta	Term	Electron microscopy	Collagen distribution and stromal alignment	Explains structural support of villi
Jirkovská et al. (2002) [7]	Human placenta	Term	Morphometric/topological analysis	Capillary network connectivity	Demonstrates organized vascular topology
McCarthy et al. (2016) [16]	Human placenta	Term and pathological	3D reconstruction	Differences in villous architecture	Provides comparative structural framework
Haeussner et al. (2016) [10]	Human placenta	Pathological (IUGR)	3D microscopy	Altered vascular branching patterns	Highlights deviations from normal structure
Mayhew et al. (2004) [18]	Human placenta	Variable	Stereology	Vascular growth and angiogenesis	Establishes baseline vascular indices
Castellucci and Kaufmann (1982) [2]	Human placenta	Term	3D structural study	Stromal architecture of villi	Defines core villous structure
Burton et al. (1996) [38]	Human placenta	Term	Stereology	Vascular adaptation to hypoxia	Functional implications of structure

Future perspectives

Computational modeling methods, together with recent advances in imaging technology, could provide new perspectives on villous architecture at the cellular and subcellular levels [39]. Branching hierarchies, capillary networks, and stromal microdomains can be studied using high-resolution 3D imaging techniques such as micro-CT, light sheet fluorescence microscopy, and multiphoton confocal imaging [39]. By analyzing stromal orientation, branching angles, capillary loop density, and distances between villi, factors that affect functional efficiency of maternal-fetal exchange, these reconstructions provide a structural foundation for quantitative assessments of villous architecture [40].

Predictive modeling of capillary perfusion dynamics and villous branching patterns is made easier by combining volumetric data with complex morphometric and topological studies. Computational simulations can establish a mechanistic knowledge of the ways in which microstructural organization influences liturgical effects by correlating certain geometric features with expected flow patterns, shear stress distributions, and transport effectiveness. Moreover, by merging spatially resolute molecular mapping with 3D ultrastructural imaging, operating microdomains within the villous tree can be recognized, such as regions with specialized nutrient transport, high metabolic activity, or significant stromal inflexibility. This effectively connects microanatomy to local natural roles.

In addition to creating uniform standards for comparison studies across gestational periods, clinical circumstances, and species, these approaches seek to improve our understanding of normative villous branching architecture. This will enable the assessment of placental remodeling and adaptability in a methodical manner. The creation of open-access digital atlases of human placental villi architecture, the standardization and development of high-resolution 3D imaging protocols, and the development of integrative analytical pipelines that combine structural, liturgical, and molecular datasets should be the top priorities of future research projects. Future studies may focus on creating open-access digital atlases, standardizing high-resolution 3D imaging protocols, and integrating structural and molecular datasets to support reproducibility, cross-study comparisons, and enhanced understanding of placental villous architecture.

## Conclusions

The present review demonstrates that branching placental villi are arranged in three dimensions to create a highly ordered structure with a distinct hierarchy. Each part, namely, the terminal, intermediate, and stem villi, plays a crucial role in maintaining the structural and functional unity of the mother-fetus bond. Branching patterns that can be seen at different stages of pregnancy indicate careful developmental control. Additionally, the intricate network of capillaries in the villi increases the area accessible for maternal-fetal exchange and facilitates effective blood circulation. The synthesis of current evidence highlights that ultrastructural modifications in the villous stroma and syncytiotrophoblast layer, such as localized thinning of the stroma, atypical capillary looping, and precise nuclear positioning, enhance diffusion rates and structural integrity during varying blood flow conditions. In high-risk conditions such as preeclampsia, intrauterine growth restriction, or maternal diabetes, these ultrastructural features are often altered, including thicker stroma, irregular capillary looping, and disrupted nuclear positioning, which may impair diffusion and maternal-fetal exchange. Collectively, these microanatomical traits define the typical architecture of healthy villi and serve as a foundation for understanding how placenta morphology supports its functions.

The ongoing use of advanced 3D imaging methods, including confocal and multiphoton microscopy, volumetric optical imaging, and quantitative morphometric analysis, is anticipated to deepen the comprehension of the intricate structure-function relationships that influence placental efficacy. This approach not only enables accurate mapping of villus shapes and capillary configurations but also provides a platform for investigating structural alterations in pathological conditions like preeclampsia, intrauterine growth restriction, and maternal metabolic disorders. Ultimately, these discoveries shift the paradigm of placental biology from mere anatomical description to predictive, function-centered models, emphasizing the hierarchical, adaptable, and biomechanically refined characteristics of the human placental chorionic tree.
